# Do Different Pretreatments of Dentine Surface Affect the Bond Strength with a Self-adhesive Resin Cement?

**DOI:** 10.3290/j.ohpd.a43355

**Published:** 2020-02-12

**Authors:** Bruna de Oliveira Reis, André Gustavo de Lima Godas, Thaís Yumi Umeda Suzuki, Ticiane Cestari Fagundes Tozzi, André Luiz Fraga Briso, Paulo Henrique dos Santos

**Affiliations:** a PhD Student, Department of Restorative Dentistry, Araçatuba School of Dentistry, São Paulo State University – UNESP, Araçatuba, São Paulo, Brazil. Established the experimental design, executed laboratorial tests and contributed to writing the manuscript.; b Research Assistant, Department of Restorative Dentistry, Araçatuba School of Dentistry, São Paulo State University – UNESP, Araçatuba, São Paulo, Brazil. Executed laboratorial tests.; c Assistant Professor, Department of Restorative Dentistry, Federal University of Minas Gerais, Belo Horizonte, Minas Gerais, Brazil. Executed laboratorial tests.; d Associate Professor, Department of Restorative Dentistry, Araçatuba School of Dentistry, São Paulo State University – UNESP, Araçatuba, São Paulo, Brazil. Established the experimental design and contributed in writing the manuscript.; e Associate Professor, Department of Restorative Dentistry, Araçatuba School of Dentistry, São Paulo State University – UNESP, Araçatuba, São Paulo, Brazil. Established the experimental design and contributed in writing the manuscript.; f Associate Professor, Department of Dental Materials and Prosthodontics, Araçatuba School of Dentistry, São Paulo State University – UNESP, Araçatuba, São Paulo, Brazil. Performed the statistical analysis.

**Keywords:** dentine-bonding agents, resin cements, tensile strength

## Abstract

**Purpose:**

To evaluate the microtensile bond strength of dentine/self-adhesive resin cement interface after several treatments on a dentine surface.

**Materials and Methods:**

Twenty-eight human molars were selected and divided into four groups: no treatment (control (C)); 2% chlorhexidine digluconate (CHX); 25% polyacrylic acid (PA); and 23 ppm dispersive solution of silver nanoparticle (SN). Prepolymerised TPH resin composite (Dentsply) blocks were luted on the dentine surface using RelyX U200 self-adhesive resin cement (3M ESPE). Microtensile bond strength was measured (MPa) in a universal testing machine 24 h and 6 months after the bonding process. The fractured specimens were examined in an optical microscope and classified according to the fracture pattern. A representative sample of each group was observed by scanning electronic microscope. Data were submitted to analysis of variance (ANOVA) and Tukey’s test to compare the mean among the groups (p <0.05).

**Results:**

The highest microtensile bond strength values after 24 h were found for the PA group (13.34 ± 6.36 MPa), with no statistically significant difference for the C group (9.76 ± 3.11 Mpa). After 6 months, the highest microtensile bond strength values were found for the C group (9.09 ± 3.27 Mpa), with statistically significant difference only for the CHX group (2.94 ± 1.66 MPa). There was statistically significant difference only for the PA group when comparing the periods studied. Regardless of the surface treatment applied, there were more adhesive failures in both periods of time.

**Conclusion:**

Dentinal pretreatment with PA, as well as use of SN before the bonding procedure of self-adhesive resin cement to dentine, may be alternative bonding protocols.

In dentistry, market demand has made many manufacturers seek more innovative forms of the development of materials with respect to aesthetics and simplicity of technical implementation. One aspect of the clinical success of indirect restorative procedures depends on the cementation technique used to establish a more stable bonding between the restoration and the various tooth structures.^24^

Due to their good mechanical properties, easy handling and aesthetic qualities, resin cements have been widely used in cementing inlays, onlays, crowns, posts and veneers. Resin cements have many advantages when compared with other cements, such as better retention, low solubility,^14^ low microleakage, and acceptable biocompatibility.^23^ Because of their bonding potential in both restoration and tooth, these materials promote reinforcements to the substrates and enable the success of restorations.^9,14^

In this context, the self-adhesive resin cements were introduced to simplify the clinical practice, and to exclude a dentinal pretreatment.^20^ These materials have acidic monomers in their composition that interact with hydroxyapatite present in the tooth, promoting micromechanical interaction and chemical adhesion to dentine.^5,7,20^ In theory, the self-adhesive cement concept is attractive because it promotes an adhesive bond to dentine covered by a smear layer without any pretreatment.^5,20^ However, a limited potential of conditioning and the ability to interact with the dentine surface was observed in some adhesive resin cements.^11,14^ Some studies^11,19^ have reported that the adhesive resin cements are not capable of forming a true hybrid layer; they only modify the smear layer producing a surface interaction at the cement–dentine interface. As a result of its low acidity, it is not very clear whether the bond strength of self-adhesive cements to dentine is affected by the quantity and/or quality of the smear layer.^11,19^

Despite statistically significant improvements, the bonding interface remains the weakest area of the restoration complex.^2^ The degradation of the resin–dentine bond region occurs over time,^11^ involving the participation of endogenous matrix metalloproteinases, making the area susceptible to hydrolytic degradation, reducing the bond strength and allowing the occurrence of infiltration.^11^ The bacterial colonisation in the oral microflora may also influence the bonding interface once substances are released during its metabolism, which may form a cariogenic biofilm causing marked demineralisation of dental tissue, thus generating changes in the microleakage and, consequently, in the bond strength.^13^

To increase the durability of the bonding interface, techniques involving dentinal pretreatment have being tested.^1,19,23^ The use of PA has been tested in order to improve the bond strength through better interaction between the resin cement and dentine surface.^19^ This acid promotes the cleaning and wetting of dentine.^19^ Besides, the use of synthetic inhibitors of matrix metalloproteinase (MMP), such as chlorhexidine digluconate (CHX)^1,3^ and SN are being used to prevent bacterial colonisation in surfaces such as catheters, prosthetics, clothing,^10^ dentine,^8,16^ root canals,^25^ and dental biofilm.^18^ However, little information is found in the literature regarding the application of solutions prior to the restorative process involving self-adhesive resin cements and dentine, especially in the long-term analysis.

Hence, the objective of this study was to evaluate the microtensile bond strength of the dentine/self-adhesive resin cement interface, after several treatments on dentine surface, in periods of 24 h and 6 months after the bonding procedure. The null hypotheses tested were: (1) different treatments performed in the dentine surface do not cause interference in the bond strength of self-adhesive resin cement to dentine; and (2) the microtensile bond strength values between the self-adhesive resin cement to dentine do not decrease after 6 months of storage compared with the values measured after 24 h of the bonding procedure.

## Materials and Methods

### Study Design

This in vitro study involved a 4 × 2 factorial study designed to evaluate the effect of different protocols of bonding to dentine of self-adhesive resin cement. The factors were pretreatment of dentine (four levels: no treatment; CHX, PA and SN), in order to test the use of ‘alternative bonding protocols’ involving the use of irrigating solutions with bacteriostatic power or modifying dentinal conditions, and two periods were introduced, 24 h or 6 months, in order to verify the effect of time in bond durability. The response variables were microtensile bond strength test (n = 7).

### Materials and Sample Preparation

The research was submitted and approved by the local Ethics Committee (#24603813.6.0000.5420). Twenty-eight freshly extracted human molars were selected, cleaned and frozen at a temperature of –20°C until the beginning of the study. All teeth with clinical evidence of caries, root resorption, cracks or fractures were excluded from the study. The materials used in the study are described in Table 1.

**Table 1:** Materials used in the study

Material	Batch #	Composition
2% chlorhexidine digluconate solution (Apothicário)	399538	2% chlorhexidine digluconate, osmosed water
25% polyacrylic acid (Riva Conditioner, SDI)	110605	25% polyacrylic acid
23 ppm dispersive solution of silver nanoparticle (Khemia)	55168	Silver nanoparticle, distilled water
RelyX U200 (3M ESPE)	491941	Base paste: glass powder treated with silane, 2-propenoic acid, 2-methyl 1,1 ‘-[1-(hydroxymetil)-1,2 ethanodlyl] esterdimethacrylate, triethylene glycol (TEG-DMA), silane-treated silica, glass fibre, sodium persulfate and per-3,5,5-trimethyl hexanoate, t-butyl. Catalyst paste: treated glass powder with silane dimethacrylate substitute silane-treated silica, sodium p-toluenesulfonate, barium 1-benzyl-5-phenyl-acid, calcium, 1,12-dodecane dimethacrylate, calcium hydroxide and titanium dioxide

The anatomical crowns of all teeth were removed through a transversal section, under water cooling, by using a low-speed diamond saw (Isomet 1000; Buehler, Lake Bluff, IL, USA). After exposure of the dentine surface, all samples were polished with #600-grit silicon carbide paper, under water cooling in an automatic polishing machine (Aropol E; Arotec Industry and Trade, Cotia, SP, Brazil) for 30 s to production of smear layer.

Teeth were randomly divided into four experimental groups (n = 7), using the lottery method, accordingly with the dentine surface treatment described: C group: no treatment of dentine surfaces was performed for this group; PA group: the dentine was etched with PA (Riva Conditioner; SDI, Melbourne, Australia) by rubbing it on the surface for 10 s. The entire surface was then washed with distilled water for 20 s and dried with absorbent paper; CHX group: CHX (Apothicário, Araçatuba, SP, Brazil) was used. With a cotton pellet, the solution was rubbed in the dentine surface for 60 s. The surface was then dried with absorbent paper; silver nanoparticle (SN) group: SN (Khemia, São Paulo, SP, Brazil) was used. Through a syringe, the dentine surface was irrigated with 3 ml of the solution. The surface was then dried with absorbent paper.

Prepolymerised blocks of TPH resin composite (Dentsply, Petrópolis, RJ, Brazil), measuring 11 mm in diameter and 4 mm in thickness – previously abraded with #600-grit silicon carbide paper, under water cooling, in an automatic polishing machine (Aropol E) – were used in the dentine/resin cement bonding procedure. The blocks were cemented directly on the dentine surface using RelyX U200 self-adhesive resin cement (3M ESPE, St. Paul, MN, USA). Prior to polymerisation, a load of 5 N was placed on the set for 3 min to standardise the thickness of the resin cement. The set was then light-cured for 20 s in all exposed surfaces using a DB 686 LED light-curing unit (light power 1200 mw/cm^2^ and potency 1.5VA) (Dabi Atlante, Ribeirão Preto, SP, Brazil). After the luting procedure, the samples were stored at 37°C for 24 h.

The samples were then cut into specimens measuring approximately 1.0 × 1.0 mm with a low-speed diamond saw under water cooling with an Isomet 1000 metallographic cutter (Buheler, Lake Bluff, IL, USA). There were five specimens for each tooth. Half of the specimens of each tooth were tested immediately after cutting, while half of the remaining specimens of each tooth were stored in artificial saliva (phosphate dibasic potassium, phosphate monobasic potassium, 70% sorbitol, sodium fluoride, potassium chloride, sodium chloride, magnesium chloride, calcium chloride, sodium benzoate, carboxymethyl cellulose, purified water) changed weekly, over 6 months.

### Microtensile Bond Strength Test

The extremities of the specimens were fixed with a cyanoacrylate adhesive (Super Bonder Gel, Henkel Corp, Rocky Hill, CT, USA) to a testing apparatus and subjected individually to microtensile testing in a OM 100 universal testing machine (Odeme Dental Research, Luzerna, PR, Brazil) at a crosshead speed of 0.7 mm/min to evaluate the microtensile bond strength (MPa). Microtensile bond strength values were obtained through the formula: force (N)/area (mm^2^).

### Optical Microscope Analysis

The fractured specimens were analysed using an optical microscope (Stemi SV 11, ZEISS, Jena, Germany) at 6× and 66× magnifications for the analysis of failure patterns and divided into four groups: (1) cohesive failure in dentine; (2) cohesive failure in the resin composite; (3) mixed failure, when involving dentine and resin composite; and (4) adhesive failure, when it occurred at the adhesive interface.

### Scanning Electron Microscopy (SEM) Analysis

A representative sample of each group was fixed in a metal stub, coated with gold sputter (Balzers SCD 050 sputter coater, Balzers, Liechtenstein), and observed by scanning electronic microscope (JEOL, JSM 5600LV, Tóquio, Japão) for the illustration of fracture patterns.

### Statistical Analysis

Microtensile bond strength data were subjected to statistical tests of normality (Shapiro–Wilk) and homogeneity of variances (Bartlett’s test). As these assumptions were accepted, data were submitted to two-way analysis of variance (ANOVA) (factor 1: pretreatment of dentine; and factor 2: period of analysis) and Tukey’s test to compare the mean among the groups (p <0.05).

## Results

The results of ANOVA (Table 2) showed that for the two study factors analysed, there is a statistically significant difference for both factors, but no statistically significant difference for the interaction between them.

**Table 2:** ANOVA table for microtensile bond strength

	DF	Sum of squares	Mean square	F value	p value	Lamda	Power
Group	3	353.282	117.761	9.666	<0.0001	28.997	0.998
Time	1	86.230	86.230	7.078	0.0106	7.0078	0.748
Group time	3	81.808	27.269	2.238	0.0958	6.715	0.524
Residual	48	584.811	12.184				

From Table 3, it is observed that the highest mean of microtensile bond strength after 24 h were found in the group PA with no statistically significant difference from the C group (p >0.05). The groups treated with SN and CHX showed a statistically significant difference when compared with the group previously treated with PA (p = 0.004). After 6 months, C group showed the highest values of microtensile bond strength with no statistically significant difference for PA as well as from SN group (p >0.05).

**Table 3:** Microtensile bond strength (MPa ± SD) of resin cement to dentine treated with different irrigating solutions after 24 h and 6 months of bonding procedure

	Control	25% polyacrylic acid	2% chlorhexidine digluconate	23 ppm silver nanoparticle
24 h	9.76 ± 3.11 A ab	13.34 ± 6.36 A	4.71 ± 2.19 A b	6.66 ± 3.56 A b
6 months	9.09 ± 3.27 A	6.73 ± 2.37 B ab	2.94 ± 1.66 A b	5.78 ± 3.27 A ab

Different letters, uppercase in the column and lowercase letters in the line, have statistically significant difference (p <0.05).

It is also observed that after 6 months the microtensile bond strength values in all groups were lower compared with the values after 24 h (Table 3). However, the difference was only statistically significant for the PA group (p = 0.024).

**Fig 1:**
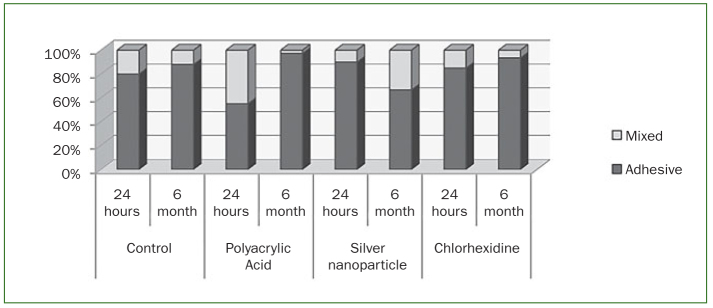
Fracture patterns (%) according to each experimental group.

The numbers of premature failures according to each group are shown in Table 4. According to the analysed failure patterns (Fig 1), there was a higher prevalence of adhesive failure, regardless of the surface treatment.

**Table 4:** Numbers of specimens with premature failure according to each group

	Control	25% polyacrylic acid	2% chlorhexidine digluconate	23 ppm silver nanoparticle
24 h	0	1	4	5
6 months	1	0	5	5

We analysed five specimens in each group.

**Fig 2:**
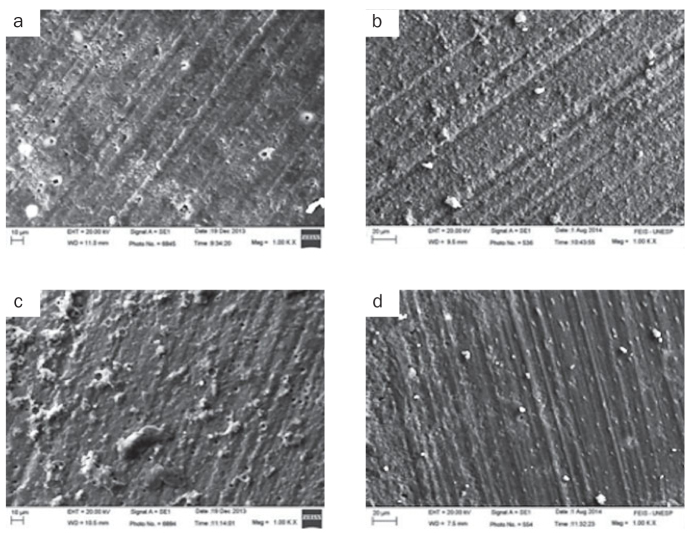
(a) Representative samples of control group fractured after 24 h. The figure shows an area with some dentine tubules exposed on the surface. (b) Representative samples of the control group fractured after 6 months. Little exposure of dentinal tubules is noted. (c) Representative samples of 25% PA group fractured after 24 h. The figure shows a modified smear layer and fragments of resin cement. (d) Representative samples of 25% PA group fractured after 6 months. Little exposure of dentinal tubules is observed.

**Fig 3:**
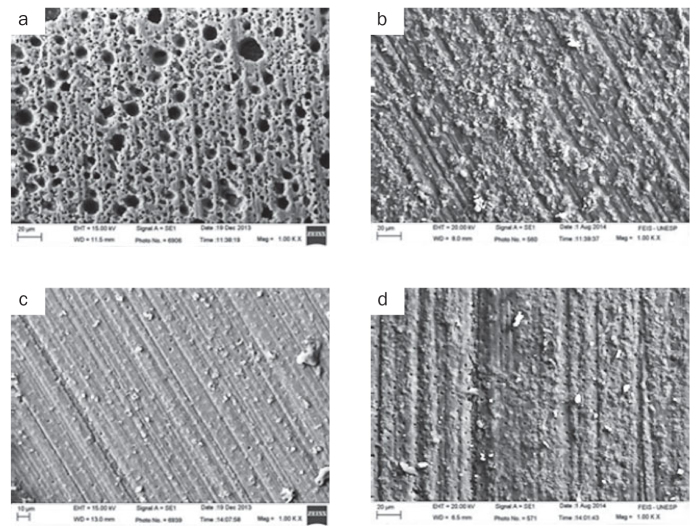
(a) Representative images of 2% chlorhexidine digluconate group fractured after 24 h. There is a non-uniform etching pattern of the dentine. (b) Representative images of the 2% chlorhexidine digluconate group fractured after 6 months. Some resin cement fragments are observed. (c) Representative images of the 23 ppm silver nanoparticle group fractured after 24 h. In the image, there is little exposure of dentinal tubules, as well as a few fragments of resin cement. (d) Representative images of the 23 ppm silver nanoparticle group fractured after 6 months. Little exposure of dentinal tubules and few resin cement fragments is observed.

Representative photomicrographs of each experimental group are displayed in Figures 2a–d and Figures 3a–d. Figures 2a and 2b are, respectively, images of the control group after 24 h and 6 months; and both show little exposure of dentinal tubules. Figures 2c and 2d are, respectively, images of the 25% polyacrylic acid (PA) group after 24 h and 6 months. After 24 h, it was observed a modified smear layer and some resin cement fragments; and after 6 months, only little exposure of dentinal tubules as in the control groups. Figures 3a and 3b, are, respectively, images of the 2% chlorhexidine digluconate group after 24 h and 6 months. After 24 h, a non-uniform etching pattern of the dentine was observed and, after 6 months, some resin cement fragments. Lastly, Figures 3c and 3d, are, respectively, images of the 23 ppm SN group after 24 h and 6 months, and in both it was possible to observe little exposure of dentinal tubules and resin cement fragments.

## Discussion

In this study, the microtensile bond strength of resin cement to the dentine surface was influenced by different pretreatments (Table 2), so the first null hypothesis of the study was rejected.

The self-adhesive resin cement, due to the high viscosity and weak acidic pH, cannot form a true hybrid layer on the dentine surface,^5,19^ which could promote lower bonding strength compared with the conventional resin cements.^11^ For the improvement of this strength, the treatment of dentine surface with acidic agents is reported in literature.^1,11,16,19,24^ In this study, the group treated with PA had the highest value of bond strength after 24 h, although with no statistically significant difference from C group (Table 3). After 6 months of bonding procedure, there was a statistically significant reduction in bond strength values for the group in which PA was used, thus rejecting the second null hypothesis of the study.

The RelyX U200, besides contain a mixture of monomers of ionised phosphoric acid, has filaments of glass-ionomer particles, corroborating some similarities with the glass-ionomer cement.^19^ Consequently, the resin cement/dentine surface adhesive interface could suffer changes in bond strength when treated with PA. PA has been used to improve the bonding of glass-ionomer cement to dentine and contains numerous carboxylic groups that could form hydrogen bonds, promote cleaning and improving the wettability of the substrate. In this study, an improvement in the bond strength values was possible, since the PA did not completely remove the smear layer, thus creating a layer rich in calcium and phosphate on the dentine surface so that then a chemical interaction between the ion and resin cement could occur.^16,19^ From the scanning electronic microscope images (Figs 2c and 2d), some resin cement fragments on the dentine surface could be observed, with partial exposure of some dentinal tubules. The exposure of the tubules is comparatively higher than the C group (Figs 2a and 2b), which could explain the higher bond strength values for the group pretreated with PA, especially 24 h after the bonding procedure.

The previous application of SN did not promote change in the bond strength values compared with the C group, at 24 h and 6 months after bonding procedure (Table 3). The scanning electronic microscope images show similar patterns of these groups (Figs 2a, 2b, 3c and 3d). A study has shown that the SN presents a relevant antibacterial potential, being widely used in medicine.^10^ In dentistry, the main use of SN is irrigating root canals.^10^ It is reported in some studies that the SN has the ability to significantly reduce bacterial colonies present within the root canals^25^ and the action on dental biofilm.^18^

The incorporation of the SN in adhesives has also been shown in some studies.^15,17,26^ A study^15^ showed that the addition of SN in the organic matrix of adhesive systems was able to reduce the metabolic activity and the production of lactic acid from *S. mutans*, the main microbial agent involved in caries formation, without causing any change in values of bond strength. For the bonding procedure of resin cements to dentine involving the prior use of SN as irrigating agent on dentine surfaces, there is still a lack of studies. However, the results of the groups previously treated with SN, which have not shown any difference from the C groups (Table 3), bring perspectives of new bonding protocols involving substances with antibacterial potential efficacy without interference in the bond strength values.

As observed in other studies,^6,12^ the lowest bond strength values were found for the groups using CHX. This values were probably the lowest because when applied to smear-covered dentine surfaces, CHX is more likely to bind to the loose apatite remnants within the smear layer and change it, than when it is applied to acid-etched dentine surfaces where, due to etching and rinsing, the phosphorate groups are depleted.^12^ The bonding mechanism of self-adhesive resin cement to tooth structure is dependent of an acidic pH of the material in the first minutes after contact with dentine.^12^ It is also reported that the interaction of the resin cement/dentine surfaces is strongly dependent on a balance in the high surface energy, mainly by the presence of calcium ions and moisture in the dentine.^6^ When the dentine surface, containing a smear layer, is treated with CHX, this could raise the pH, creating precipitates in contact with phosphate ions from hydroxyapatite of the dentine surface.^6^ A reduction in the ratio of phosphate/calcium ions could reduce the bonding potential of resin cements, with consequent reduction in the bond strength of these cements to dentine surface.^6^ From Figures 3a and 3b, there is a non-uniform etching pattern of dentine, which could have contributed to the lower bond strength values found in this group. Although there was a reduction in the bond strength of the groups treated with CHX (in both tests: after 24 h and 6 months of bonding procedure), further long-term studies are needed. Some studies have shown the characteristic of CHX as a synthetic inhibitor of matrix metalloproteinases (MMPs) responsible for the degradation of the hybrid layer.^1^ Other studies are still needed to complement this information.

The classification given when interpreting the failure mode is important and requires very careful consideration. Cohesive failure in dentine or resin can occur with the microtensile bond test due to errors in the alignment of the specimen along the long axis of the testing device^4,22^ or from the introduction of microcracks during cutting or trimming of the specimens.^21,22^ Also, mixed failures represent breaking stresses derived from different materials with different mechanical properties and thus no longer are representative of adhesive modes of failure.^22^ In this study, the main failure mode found for the self-adhesive cement tested, in all groups, was adhesive failure at the cement–dentine interface (Fig 1), showing that the true adhesive interface was tested.^22^ The same was also observed in other studies testing self-adhesive resin cements.^19,24^

In relation to the clinical implications of this study, it is known that the resin–dentine bond interface degrades over time and is considered the weakest area of the restoration complex, compromising the clinical prognosis. Thus, alternative bonding protocols seem to be viable in the improvement of the bond strength through a better interaction between the resin cement and dentine surface.

## Conclusion

It was possible to conclude that in the bonding procedure of self-adhesive resin cement to dentine, the dentinal pretreatment with PA, as well as the use of SN before the bonding procedure, showed results with no difference compared with the C group. Some limitations, such as the short period of ageing and the evaluation of just one resin cement in the bonding procedure, may be considered.
